# “BRUSH UP”: AN INNOVATIVE TECHNOLOGICAL AID FOR PARENTS TO KEEP A CHECK OF THEIR CHILDREN’S ORAL HYGIENE BEHAVIOUR

**DOI:** 10.1590/1984-0462/2021/39/2020085

**Published:** 2021-04-02

**Authors:** Ruttika Vijay Desai, Nivedita Chandrashekhar Badrapur, Harshitha Mittapalli, Bagepalli Keshavappa Srivastava, Shruthi Eshwar, Vipin Jain

**Affiliations:** a Dr. D.Y. Patil Vidyapeeth, Pune, Maharashtra, India.; b P.M.N.M. Dental College and Hospital, Bagalkot, India.; c K.L.E Society’s Institute of Dental Sciences, Bangalore, India.

**Keywords:** Mobile application, Smartphone, Toothbrushing, Dental plaque, Aplicativos móveis, Smartphone, Escovação dentária, Placa dentária

## Abstract

**Objective:**

To investigate the impact of “Brush Up” - a mobile application, on oral hygiene behaviours of 4-6-year-old children in Bangalore city.

**Methods:**

In this experimental study, 247 children aged 4-6, were randomly divided into three groups. Considering “Brush Up” is a mobile application, parents of the children in Group 1 (n=82) downloaded the application on their smartphones. Children in Group 2 (n=83) and Group 3 (n=82) received tooth brushing instructions by an educative video and manual demonstration, respectively. Effectiveness of tooth brushing was assessed with plaque scores, which were recorded for all the groups at baseline and one month using Visible Biofilm Index.

**Results:**

Wilcoxon signed rank test showed a significant improvement in the tooth brushing behaviour for the Brush Up group, which was indicated by a lower plaque score after a follow-up of one month. Kruskal-Wallis test followed by *post-hoc* test showed that the mean ranks of plaque scores of Brush Up group are consistently lower than those of video demonstration group and manual demonstration group.

**Conclusions:**

The lower plaque score in subsequent follow-up in Brush Up group suggests that using a smart system can enhance learning a correct tooth brushing method in young children and can also help in implementing the required reinforcement and motivation to brush and aid in better plaque control.

## RESUMO

**Objetivo:**

Investigar o impacto do “Brush Up”, um aplicativo móvel, sobre comportamentos de higiene bucal de crianças de 4 a 6 anos na cidade de Bangalore, na Índia.

**Métodos:**

Neste estudo experimental, 247 crianças de 4 a 6 anos foram divididas aleatoriamente em 3 grupos. Como “Brush Up” é um aplicativo móvel, os pais das crianças do Grupo 1 (n=82) baixaram o aplicativo em seus smartphones. As crianças do grupo 2 (n=83) e do grupo 3 (n=82) receberam instruções sobre escovação por meio de vídeo educativo e demonstração manual, respectivamente. A efetividade da escovação foi avaliada por meio de escores de placa bacteriana, registrados para todos os grupos antes da intervenção e um mês depois.

**Resultados:**

O teste de Wilcoxon sinalizou uma melhora significativa na escovação para o grupo “Brush Up”, com uma pontuação mais baixa na placa bacteriana após um mês de acompanhamento. O teste de Kruskal-Wallis, seguido do teste post hoc, mostrou que as classificações médias das pontuações do grupo “Brush Up” foram consistentemente mais baixas do que as do grupo com demonstração em vídeo e demonstração manual.

**Conclusões:**

Os resultados obtidos no grupo “Brush Up” sugerem que o uso de um sistema inteligente pode melhorar o aprendizado de um método correto de escovação em crianças pequenas e ajudar na implementação do reforço e motivação necessários para escovar e controlar melhor a placa bacteriana.

## INTRODUCTION

The role of microbial plaque in the initiation and progression of dental caries, and periodontal disease is well documented. Mechanical removal of plaque at regular intervals currently offers the safest mode of preventive therapy.^1^ Thus, tooth brushing continues to be a widely used and effective method for cleaning most of teeth surfaces.^2^

Tooth brushing is acquired during the social learning process of children. When taught in early childhood, it gets naturally ingrained in their daily routine, and only positive reinforcement is needed later.^2^ Improper tooth brushing behaviors can result in gingivitis, tooth decay, or, eventually, tooth loss. The primary goal of any brushing technique is to reach all accessible teeth surfaces and enable patients to prevent trauma from brushing.^3^

From all the known brushing techniques, the Modified Bass Technique (MBT) significantly removes supragingival plaque, thereby improving the level of oral hygiene, with little soft tissue damage observed.^3^ Studies report that children have problems with grammatical understanding at their early age, which is reflected in their difficulty to understand verbal instructions regarding tooth brushing given to them in schools. To overcome this barrier, audio-visual aids have been proven to be effective.^2^

Encouraging new positive behaviors in young children is vital, but turning them into habits becomes a challenge for parents and teachers. As a result, play-based occupational therapy has been developed to induce behavioral change.^4^ Interventions involving automated message systems have shown to improve knowledge and health outcomes in a variety of health areas. Technologies such as cell phones and text messaging, which are already a part of people’s daily lives, have a great potential for improving health by assisting them with behavior modification and disease self-management.^5^

Mobile phones have had a considerable impact in developing countries. Across the world, people are gaining access to the internet via mobile phones.^6^ The number of smartphone users in India alone was found to be about 502.2 million in 2019,^7^ projected to increase to 829 million by 2022.^8^ Mobile health (mHealth) is the use of mobile phone technology to deliver health care. Mobile phone technologies that have been used for mHealth include text messaging, video messaging, voice calling, and internet connectivity.^6^

The average 5-year-old child brushes only 25% of all areas of teeth. Considering young children are often deficient in brushing their teeth thoroughly and uniformly, supervision of parents and/or teachers is needed to teach them proper brushing techniques.^4^ Thus, the use of smartphones to promote oral health seems like an interesting and feasible option. Gaming apps running on mobile devices are useful means to deliver health interventions and motivate self-care. Research into the use of apps is starting to emerge in healthcare, but in the oral health area, research is minimal.^9^ Thus, the present study aims to investigate the impact of a mobile application (Brush Up) on oral hygiene behaviors of 4-6 year old children in Bangalore City.

## METHOD

This study was conducted with children aged from 4 to 6, attending a private school in Bangalore City, India. Convenience sampling technique was used for selection of study subjects. The study received ethical approval by the institutional review board of the KLE Institute of Dental sciences, Bangalore [KIDS/IEC/NOV-2017/5]. The study was conducted between December 2017 and January 2018. Written informed consent was collected from the school authorities and parents, and verbal consent was obtained from the subjects. Only the subjects who matched the age group of 4-6, who were present on the day of examination, and whose parents were smartphone users were included in the study. Out of 305 subjects, 247 were included in the study.

Before commencing the study, the chief investigator was trained in the Department of Public Health Dentistry, K.L.E Society’s Institute of Dental Sciences, Bangalore, to record plaque score using Modified Visible Biofilm Index.^10^ ^,^ ^11^ *Kappa* coefficient value (ƙ) for intra-examiner reliability was 0.89. A self-designed format was used to record data regarding general information (age, gender, frequency of tooth brushing, duration of tooth brushing, cleaning of the lingual surfaces of teeth, and tongue cleaning). This was followed by recording the plaque scores.

After interviewing, the subjects were randomly divided into three groups. Randomization was performed by one investigator, using random number table, whereas another investigator assigned the participants into the three intervention groups. *Group 1* (Brush Up Group, n=82) received tooth brushing instructions using the mobile application ‘Brush Up’. Brush Up is a ‘toothbrush training game’ application developed by GamesThatWork. It has a cartoon toothbrush tutor called ‘BUDD’, that motivates children to brush their teeth with him. The app shows BUDD cleaning all the teeth surfaces and it makes use of the phone’s front camera so that children can repeat the action and watch themselves while brushing to make sure they are doing it right. The app also uses an instructional song during the exercise, which lasts approximately three minutes.^12^ Thus, children stop their brushing routine when BUDD stops at the end of the three minutes. The application is freely available on Google play store. The link to it was shared with the parents, and they downloaded the application in the presence of an intern from the Department. Parents were instructed to use the application twice a day for one month. *Group 2* (Video demonstration group, n=83) received tooth brushing instructions by an educational video distributed to teachers. The teachers played the video for these subjects after the 1^st^ clinical examination. *Group 3* (Manual demonstration group, n=82) was given tooth brushing instructions with manual demonstration, using a model and toothbrush by another trained intern after the 1^st^ clinical examination. Groups 2 and 3 were given instructions in separate classrooms. All the subjects were examined by the chief investigator for plaque status using Modified Visible Biofilm Index^10^ ^,^ ^11^ at baseline and after one month follow up to assess the effectiveness of the education provided. The instruments used to assess plaque status were mouth mirror, blunt probe, and gauze for drying the teeth. All the investigators performed specific tasks, and blinding was done at each stage. All the teeth surfaces were examined, and teeth were scored accordingly.

Data was analysed using IBM SPSS v.20. Descriptive statistics were computed to summarize the characteristics of the subjects. Since the data was not normal as tested by the Kolmogorov-Smirnov test, Wilcoxon Signed Rank test evaluated the effect of mobile application on toothbrushing behaviours in the Brush Up group using the baseline and follow-up plaque scores. Kruskal-Wallis ANOVA was used for inter-group comparison. Mann-Whitney U test was used as a *post-hoc* test.

## RESULTS

A total of 247 subjects participated in the study. Out of these 80 (32.4%) belonged to the 4 year age group, 88 (35.6%) to 5 year age group and 79 (32%) to 6 year age group. The mean age of the subjects was 4.98 years (SD±0.84). The sample consisted of 126 (51%) males and 121 (49%) females. At baseline, only 80 (32.4%) subjects brushed ≥2times a day, and 184 (74.5%) reported not brushing the lingual teeth surfaces. At follow up of 1 month, a radical improvement was observed, with 166 (67.2%) subjects reporting brushing ≥2times a day and only 74 (30%) reporting about not brushing the lingual surfaces of teeth (Table 1).

**Table 1:** Baseline characteristics of participants.

Characteristics	Frequency	Percentage
Age in years old		
4	80	32.4
5	88	35.6
6	79	32.0
Gender		
Male	126	51.0
Female	121	49.0
Baseline *versus* Follow up		
Frequency of tooth brushing		
Baseline		
Once	167	67.6
≥2 times	80	32.4
Follow-up		
Once	81	32.8
≥2 times	166	67.2
Duration of tooth brushing		
Baseline		
<2mins	118	47.8
≥2mins	129	52.2
Follow-up		
<2mins	84	34.0
≥2mins	163	66.0
Cleaning of lingual teeth surfaces		
Baseline		
Yes	63	25.5
No	184	74.5
Follow-up		
Yes	173	70.0
No	74	30.0
Tongue cleaning		
Baseline		
Yes	150	60.7
No	97	39.3
Follow-up		
Yes	208	84.2
No	39	15.8
Plaque levels-		
Baseline		
No visible plaque	0	0
Thin plaque	225	91.1
Thick plaque	22	8.9
Follow-up		
No visible plaque	57	23.1
Thin plaque	190	76.9
Thick plaque	0	0

Chi-square test revealed that, there was a significant change in the frequency and duration of toothbrushing, cleaning of the lingual surfaces of teeth and tongue cleaning for all groups, post education. However, for Brush Up group*,* the scores for tongue cleaning did not change much when compared to the other two groups (Table 2). Wilcoxon signed rank test (Table 3) showed that the follow-up plaque score was lower than baseline score for 96.3% of the subjects, and the plaque score was equal for 3.7% of the subjects (p=0.001) indicating a significant improvement in the oral hygiene.

**Table 2:** Comparison of oral hygiene habits pre- and post-intervention.

Characteristics	Group 1 (Mobile Application)	Group 2 (Video Demonstration)	Group 3 (Manual Demonstration)	p-value
Frequency of tooth brushing (brushing twice daily)				
PRE intervention	33 (40.2%)	33 (39.8%)	14 (17.1%)	0.003
POST intervention	67 (81.7%)	50 (60.2%)	49 (59.8%)	
Duration of tooth brushing (2-4 mins)				
PRE intervention	30 (36.6%)	39 (47%)	28 (34.1%)	<0.001
POST intervention	71 (86.6%)	58 (69.9%)	33 (40.2%)	
Cleaning of lingual surfaces (those who responded “Yes”)				
PRE intervention	21 (25.6%)	34 (41%)	08 (9.8%)	<0.001
POST intervention	78 (95.1%)	57 (68.7%)	38 (46.3%)	
Tongue cleaning (those who responded “Yes”)				
PRE intervention	56 (68.3%)	61 (73.5%)	33 (40.2%)	<0.001
POST intervention	57 (69.5%)	79 (95.2%)	72 (87.8%)	

**Table 3:** Comparison between baseline and follow up plaque scores for the Brush Up group.

Follow up - Baseline	Ranks	n	Mean rank	p-value
Group 1 (Brush Up)	Negative	79^a^	40	0.001
	Positive	0^b^	0	
	Ties	3^c^		

^a^Follow up score<baseline score; ^b^follow up score>baseline score; ^c^follow up score=baseline score.

The comparison of plaque scores between the three groups at baseline and at follow up showed a statistically significant difference for plaque scores at follow up (p*=*0.001). The mean rank for Brush Up group was significantly lower when compared to the other groups, suggesting lower plaque scores at follow up (Table 4). *Post-hoc* analysis using Mann-Whitney U test showed that there was a significant difference between plaque levels of all groups; however, the mean rank for Brush Up group was consistently low (Table 4).

**Table 4:** Comparison between different methods of tooth brushing instructions.

	Group	n	Mean rank	p-value
VBI-Baseline*	Brush Up app (G1)	82	122.5	0.052
	Video demo (G2)	83	114.8	
	Manual demo (G3)	82	134.8	
	Total	247		
VBI-Follow up	Brush Up app (G1)	82	70.9	<0.001
	Video demo (G2)	83	122.5	
	Manual demo (G3)	82	178.6	
	Total	247		
Follow up**	Brush Up app (G1)	82	63.8	0.001
	Video demo (G2)	83	102	
	Total	165		
	Brush Up app (G1)	82	48.7	0.001
	Manual Demo (G3)	82	116.3	
	Total	164		
	Video Demo (G2)	83	62.5	0.001
	Manual Demo	82	103.8	
	Total	165		

VBI: Visible Biofilm Index; *Kruskal Wallis ANOVA test; **Mann-Whitney U as a *post-hoc* test.

## DISCUSSION

The key feature of any mobile health app is its ability to provide social and clinical support for the consumer, and documentation of self-administered readings and symptoms. This provides greater insights into the control and management of medical conditions, and enables the healthcare providers as well as the consumers for the same.^13^ Because of limited published literature on the use of health apps specifically for oral care; this remains a relatively new research area.

It is already known that mechanical plaque control, despite being the most effective method, is inefficient in young children under 10 years old, due to poor manual dexterity and lack of sufficient motivation.^14^ ^,^ ^15^ Thus, instructions should be given according to the child’s degree of readiness for tooth brushing, and should include systematic training and reinforcement. Although manual dexterity and ability are needed, intensive individual training is essential, and children should be educated in oral self-care according to the status of their psychological development.^2^

Since our study population was 4-6-year old children, the parents/guardians supervised their tooth brushing routine for all the groups. Teaching tooth brushing and turning it into a habit require that parents create a routine that includes helping their children develop the necessary skills.^16^ The present study made use of a freely available android application for tooth brushing, called “Brush Up”, which uses this concept by creating an entertaining environment to engage children and motivate them to incorporate tooth brushing into their routine of daily habits.^12^

Fedele conducted a meta-analysis to determine the effectiveness of mHealth interventions in improving health-related outcomes in young children. Their results indicated that mHealth interventions can be effective in producing meaningful health behavior and associated health outcomes in pediatric patients; when incorporated with parental supervision, the results were more effective than when seen with the pediatric patient alone.^17^

 Health education is a time-tested, well accepted and effective way to enhance oral hygiene procedures, and prevent plaque associated oral diseases.^18^ Our findings suggest that all health education aids work positively towards improving tooth brushing behaviors. However, the performance of the mobile application group was consistently better than the other groups in everything, except tongue cleaning. Significant improvements in the scores were observed in the Brush Up group from baseline to follow-up for frequency of tooth brushing, duration of tooth brushing, and cleaning of lingual surfaces. This may be because the children watched BUDD, that brushes alongside them on the screen. There is only one published pilot study, conducted by Jacobson et al., that found similar results.^12^ This is the only study which used “Brush Up” application, to which the results of our study can directly be compared. They found that there was a 61.8% increase in brushing lingual surfaces by children using the app at seven-day follow-up, which increased to 93.3% at 14-day follow-up, which is in agreement with our findings (25.60% at baseline, and 95.12% at one month follow-up). However, for tongue cleaning, there were minimal changes at follow-up. This may be attributed to the lack of tongue cleaning exercise in the tooth brushing routine of the mobile app. The other groups fared better in the tongue cleaning exercise, because regular health education instruction includes explanation of tongue cleaning as one of the important steps for maintaining good oral hygiene. At one-year follow-up without the use of app, Jacobson et al. found that the improvements were not statistically significant, which they attributed to the lack of re-enforcement of the health education. In our study, this re-enforcement can explain the consistent improvement seen in Brush Up group when compared to other groups at one-month follow-up for all factors (with the exception of tongue cleaning), suggesting that routinely using the mobile application for tooth brushing may have inculcated a positive habit that led to the improvement in oral hygiene. Given there was no further follow-up, it is safe to assume that further re-enforcement can lead to sustained maintenance of good oral hygiene.

There are other studies in Orthodontics and other healthcare sciences that used mobile applications and other mobile health interventions and may be used for the comparison of our results to some extent. A study conducted by Alkadhi et al. on 12-year old children undergoing fixed orthodontic treatment, found that the Plaque Index and Gingival Index scores showed significant reductions at four-week follow-up for the group that received mobile application instructions (self-designed application which included video oral hygiene instructions and reminders with push notifications) when compared to the group that received verbal oral hygiene instructions at every visit.^19^ This study may be used for comparison considering there is difficulty in practicing proper oral hygiene with the appliance on and decreased compliance by the patients, which is comparable to some extent, to the poor manual dexterity, poor attitudes, and behavior towards oral hygiene among young children aged 4-6. Our findings suggest that the improvement in oral hygiene was better for the Brush Up group, which was followed by the video-demonstration group. This is in accordance with studies conducted by Graetz et al.,^3^ Underwood et al.^9^, and Alkadhi et al.,^19^ who showed that using a smart system to monitor tooth brushing, resulted in more thorough brushing across the dentition because of prolonged learning, leading to an improvement in oral hygiene.

The strength of this study was the reinforcement of the oral hygiene instruction provided daily with the mobile application, which is practically not feasible using the other instruction techniques. Despite promising results, the study had several limitations. The present sample of participants is not representative, because a convenience method of sampling was employed, which provides only partial generalizability. The follow-up period was short, and we recommend future studies with longer follow-up periods to confirm the findings. Because the inclusion criteria required parents using smartphones, the results are restricted to smartphone users. The mobile application used makes use of modified bass technique, which is a complex technique to master, especially by young children. The application only demonstrates tooth brushing, and does not demonstrate tongue cleaning, which is an important part of oral hygiene.

Within the study limitations of, the Brush Up application via mobile technology showed improvement in oral hygiene behaviors of children and can help parents in keeping a check on their children’s hygiene behavior. Moreover, an interactive gaming application can be used to spark interest and motivation regarding tooth brushing in young children. A mobile application is a promising tool to motivate an evidence-based oral hygiene routine by helping in behavioral change, skill development, and, thus, turning an exercise into a habit.
